# A Young Man with Chest Pain 

**DOI:** 10.24908/pocus.v9i2.17500

**Published:** 2024-11-15

**Authors:** Anderson Wang, Aalap Shah

**Affiliations:** 1 Department of Emergency Medicine, Medical University of South Carolina Charleston, SC USA

**Keywords:** POCUS, Gallbladder, Emergency Department, Emergency Medicine

## Abstract

Acalculous cholecystitis is a life-threatening diagnosis that is more commonly associated with ill patients in the ICU. We present a case of acute acalculous cholecystitis (ACC) in an otherwise healthy 18-year-old man who presented to the Emergency Department (ED) with right-sided chest pain that was ultimately diagnosed with point of care ultrasound (POCUS). This case demonstrates the importance of conducting a thorough history and physical as well as the importance of POCUS to aid in clinical decision making and its value in diagnosing acute biliary pathology in the ED.

## Case Presentation

**Figure 1:**
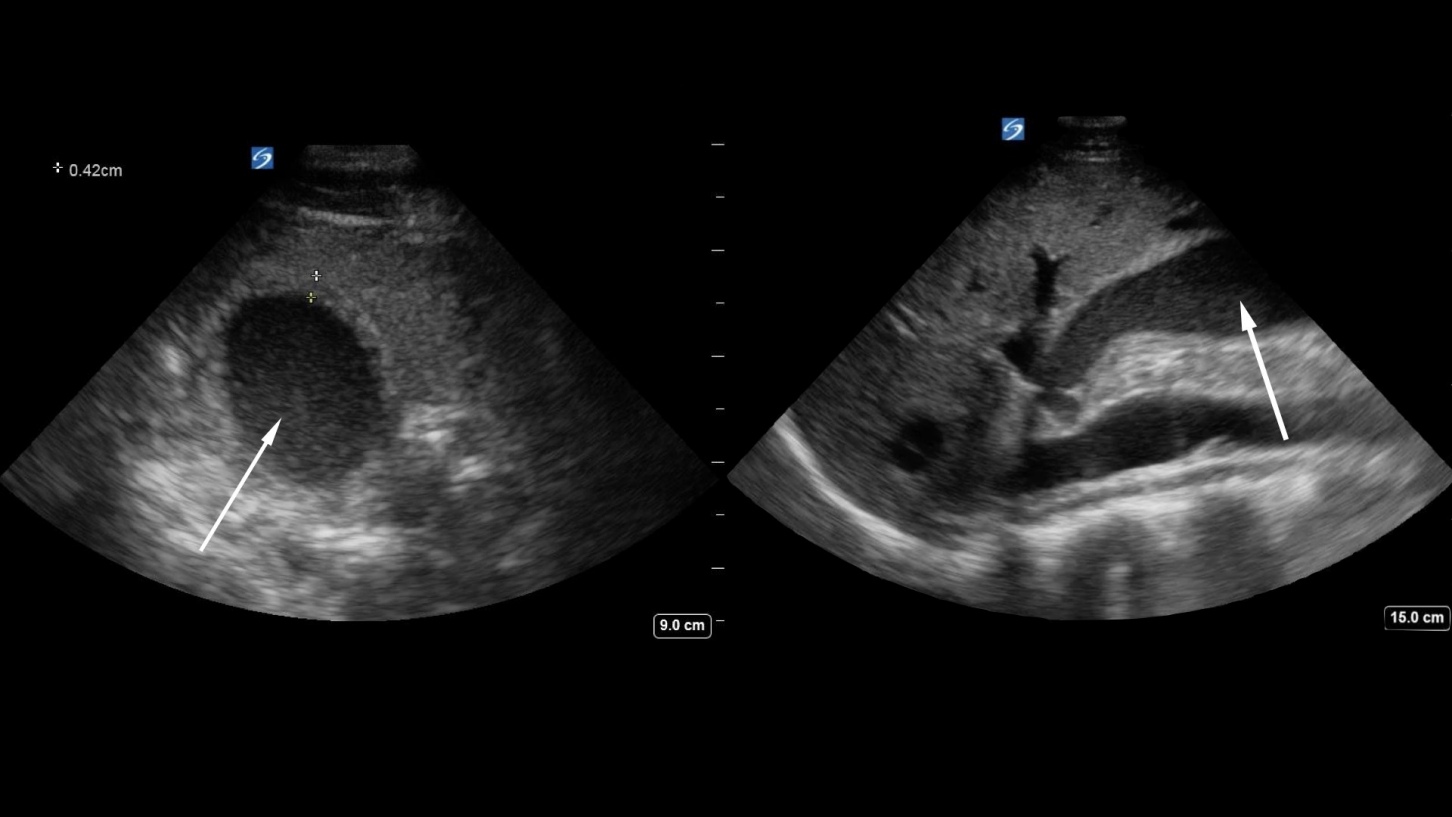
Left Pane: Ultrasound short axis view of gallbladder. Gallbladder is distended with sludge material within (Arrow). Anterior wall of gallbladder measured at 0.42cm (Calipers). Right Pane: Ultrasound long axis view of gallbladder. Gallbladder is distended with sludge material within (Arrow). No gallstones noted.

An 18-year-old man presented initially for the complaint of right-sided chest pain. He had no past surgical history and had a past medical history significant only for anxiety and asthma. The patient described his symptoms as a sensation of “being punched in the chest.” He also reported epigastric pain that had been recurrent 4-5 times in the past month, primarily in the evenings, typically lasting 20-30 minutes in duration. He denied having any fever, chills, nausea, vomiting during these episodes, or currently. He had previously seen his primary care physician for these symptoms and had been diagnosed with presumed gastritis and gastroesophageal reflux. As a result, he was prescribed a treatment trial on omeprazole, although without success. The patient reported his symptoms were getting more severe and persistent, which prompted an ED visit. On arrival, he was tachycardic with a heart rate of 112, mildly hypertensive with blood pressure 148/91, had an oral temperature of 36.9C, and was saturating 99% on room air with unlabored respirations of 16. His physical exam was notable for diffuse abdominal tenderness that was worse in the epigastric region. Based on the physical exam, the ED physician performed a right upper quadrant POCUS examination (Figure 1 and Video S1).

## Diagnosis

### Acute Acalculous Cholecystitis

POCUS demonstrated a positive sonographic Murphy’s sign, biliary sludge, and a thickened, edematous gallbladder wall with and a normal common bile duct diameter and absence of gallstones. The patient was given piperacillin-tazobactam, intravenous fluids and pain medication. General Surgery was consulted, and the patient was admitted and underwent a routine laparoscopic cholecystectomy the following afternoon. Notably, according to intraoperative documentation, the procedure was complicated by a difficult view of the Triangle of Calot as the cystic artery was obstructed by a large overlying lymph node. The cystic duct was kinked off by chronic adhesions between the infundibulum and cystic duct. The patient was subsequently discharged later that day**. **The patient followed up with the general surgeons in clinic and was doing well without any complications.

ACC is a time-sensitive diagnosis that can result in severe outcomes such as neurological illness or even death. It is often caused by bile stasis or ischemia, which can be caused by systemic inflammation, systemic illness, or obstruction 1, 2, 3. In this case, the patient likely developed AAC in the absence of traditional risk factors secondary to previously undiagnosed adhesions and biliary tree obstruction, as noted in the operative note. While this is a rare presentation, it emphasizes the importance of maintaining a high index of suspicion for biliary disease. It also shows the utility of POCUS, which has comparable test characteristics compared to radiology performed ultrasound, while avoiding strain on additional systems resources and avoiding additional radiation exposure 4, 5, 6. A recent meta-analysis of emergency physician performed biliary POCUS revealed a pooled sensitivity of of 70.9% and specificity of 94.4% 4. In this case, rapidly performed POCUS at the bedside was able to immediately narrow the diagnosis within minutes, leading to expedited management and avoidance of additional unnecessary testing.

## Patient Consent 

Verbal informed consent was obtained for the purpose of scientific and academic divulgation.

## Disclosure Statement

The authors do not report any conflict of interest. [AW,AS] have nothing to disclose. 

## Supplementary Material

Video S1 Video S1 Right upper quadrant POCUS examination.
